# Genomic Epidemiology of Carbapenemase-producing *Klebsiella pneumoniae* in China

**DOI:** 10.1016/j.gpb.2022.02.005

**Published:** 2022-03-18

**Authors:** Cuidan Li, Xiaoyuan Jiang, Tingting Yang, Yingjiao Ju, Zhe Yin, Liya Yue, Guannan Ma, Xuebing Wang, Ying Jing, Xinhua Luo, Shuangshuang Li, Xue Yang, Fei Chen, Dongsheng Zhou

**Affiliations:** 1 CAS Key Laboratory of Genome Sciences & Information, Beijing Institute of Genomics, Chinese Academy of Sciences and China National Center for Bioinformation, Beijing 100101, China; 2 State Key Laboratory of Pathogen and Biosecurity, Beijing Institute of Microbiology and Epidemiology, Beijing 100071, China; 3 University of Chinese Academy of Sciences, Beijing 100049, China; 4 State Key Laboratory of Pathogenesis, Prevention and Treatment of High Incidence Diseases in Central Asia, Urumqi 830011, China; 5 Beijing Key Laboratory of Genome and Precision Medicine Technologies, Beijing 100101, China

**Keywords:** *Klebsiella pneumoniae*, Drug resistance, Carbapenemase, Plasmid, Genomic epidemiology

## Abstract

The rapid spread of **carbapenemase**-producing ***Klebsiella pneumoniae*** (cpKP) poses serious threats to public health; however, the underlying genetic basis for its dissemination is still unknown. We conducted a comprehensive **genomic epidemiology** analysis on 420 cpKP isolates collected from 70 hospitals in 24 provinces/autonomous regions/municipalities of China during 2009–2017 by short-/long-read sequencing. The results showed that most cpKP isolates were categorized into clonal group 258 (CG258), in which ST11 was the dominant clone. Phylogenetic analysis revealed three major clades including the top one of Clade 3 for CG258 cpKP isolates. Additionally, carbapenemase gene analysis indicated that *bla*_KPC_ was dominant in the cpKP isolates, and most *bla*_KPC_ genes were located in five major incompatibility (Inc) groups of *bla*_KPC_-harboring plasmids. Importantly, three advantageous combinations of host–*bla*_KPC_-carrying plasmid (Clade 3.1+3.2–IncFII_pHN7A8_, Clade 3.1+3.2–IncFII_pHN7A8_:IncR, and Clade 3.3–IncFII_pHN7A8_:Inc_pA1763-KPC_) were identified to confer cpKP isolates the advantages in both genotypes (strong correlation/coevolution) and phenotypes (resistance/growth/competition) to facilitate the nationwide spread of ST11/CG258 cpKP. Intriguingly, Bayesian skyline analysis illustrated that the three advantageous combinations might be directly associated with the strong population expansion during 2007–2008 and subsequent maintenance of the population of ST11/CG258 cpKP after 2008. We then examined **drug resistance** profiles of these cpKP isolates and proposed combination treatment regimens for CG258/non-CG258 cpKP infections. Thus, the findings of our systematical analysis shed light on the molecular epidemiology and genetic basis for the dissemination of ST11/CG258 cpKP in China, and much emphasis should be given to the close monitoring of advantageous cpKP–**plasmid** combinations.

## Introduction

Clinical carbapenem-resistant *Klebsiella pneumoniae* (KP) isolates spread rapidly worldwide in recent years, posing a serious health threat due to their high morbidity and mortality rates, limited treatment options, prolonged hospitalization, and high treatment costs [1], [2], [3], [4], [5]. The production of carbapenemases is one of the major causes of carbapenem resistance in KP, and carbapenemase-producing KP (cpKP) has emerged as a threatening epidemic pathogen in hospital settings [2], [6]. The carbapenemase genes in cpKP strains mainly include *bla*_KPC_, *bla*_NDM_, and *bla*_IMP_, of which *bla*_KPC_ is the most clinically frequent in most countries [2], [7]. They are typically carried in the plasmids of many incompatibility (Inc) groups such as IncFII, X, I, C, N, R, P-2, U, W, and L/M [2], [8].

Genomic studies have shown that the clonal group CG258 is closely associated with cpKP isolates, especially *bla*_KPC_-carrying cpKP isolates [9], [10], [11], [12], [13]. CG258 mainly consists of ST258 and its single-locus allelic variants ST11 and ST512. The cpKP isolates of ST258 and ST512 are mostly prevalent in American and European countries [8], [11], [14], whereas those of ST11 are highly dominant in China [15], [16], [17].

Although 115 sequencing types (STs) of cpKP isolates have been identified worldwide so far [8], it is reported that only ST11/CG258 has successfully disseminated in China [15], [16], [17]. The causes for this phenomenon are still unclear up to date. Therefore, a large-scale genomic study on cpKP isolates is necessary to uncover the genetic basis for its dissemination in China.

In this study, we employed short-/long-read sequencing technologies to comprehensively analyze the genomic epidemiology in 420 clinical cpKP isolates collected from multicenter hospitals of 24 Chinese provinces/autonomous regions/municipalities from 2009 to 2017. The results depicted a population snapshot of cpKP isolates harboring mainly *bla*_KPC_-carrying plasmids of diverse Inc groups and further provided insights into the evolution of host KP-*bla*_KPC_-carrying plasmids and their roles in the nationwide spread of ST11/CG258 cpKP isolates.

## Results

### Genetic diversity of CG258 and non-CG258 cpKP isolates in China

**Figure 1:**
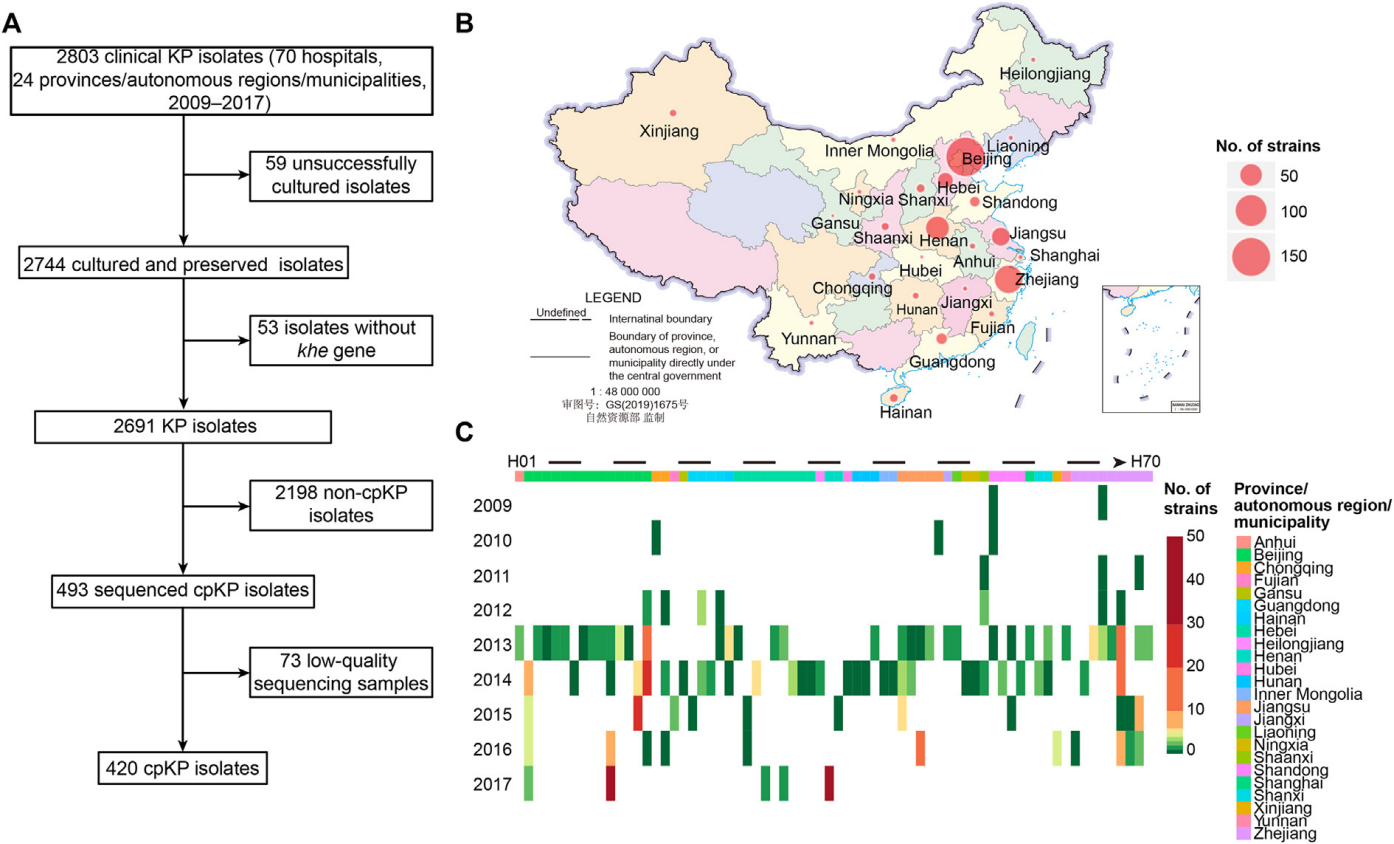
**Spatial-temporal distribution of cpKP isolates from China** **A.** Screening process of the 420 cpKP isolates. **B.** Distribution of our 420 cpKP isolates in different provinces/autonomous regions/municipalities. **C.** Distribution of the 420 cpKP isolates in different years and hospitals. Hospital H1 to H70 could be assigned to different provinces/autonomous regions/municipalities in different colors. cpKP, carbapenemase-producing *Klebsiella pneumoniae*.

**Supplementary figure S1:**
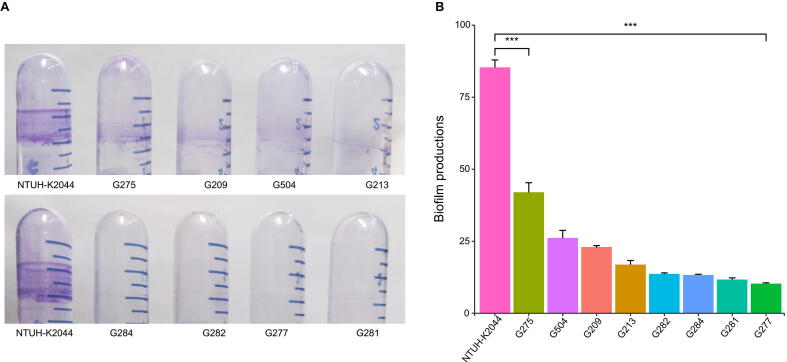
**Biofilm formation assay for the cpKP isolates**. **A**. Side views of the test tubes with biofilms at 24 h after crystal violet staining. Eight isolates (namely G275, G209, G504, G213, G284, G282, G277, and G281) were randomly selected from our 420 cpKP isolates, and NTUH-K2044 was included as positive control. **B**. Quantitative calculation of biofilm product for the isolates at the 24 h post staining. The biofilm production was calculated by 1000×*OD_570_*/(*OD_600_*×V1×V2); V1 refers to the volume of initial bacterial solution, and V2 refers to the volume of DMSO.

A total of 420 cpKP isolates from 70 hospitals of 24 Chinese provinces/autonomous regions/municipalities obtained during the period 2009–2017 were sequenced using the Illumina sequencing platform, and 69 of them were further sequenced for complete genomes using the PacBio (35/69) or Nanopore (34/69) sequencing platforms (Figure 1; Table S1). Virulence gene analysis (Table S2) and biofilm formation experiments (Figure S1) demonstrated the multidrug resistant phenotype with low virulence in cpKP isolates, which is consistent with the previous report [8].

**Figure 2:**
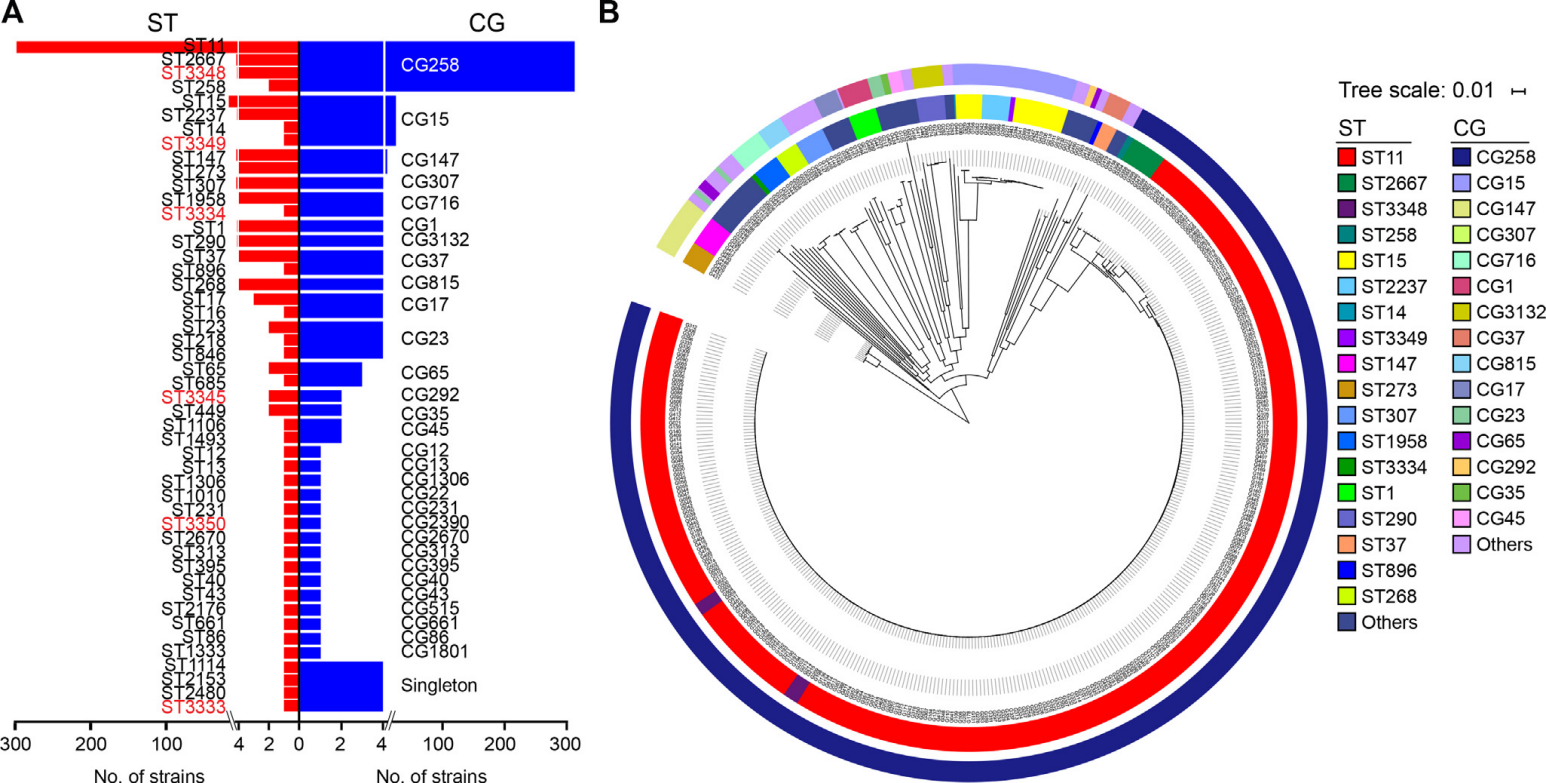
**MLST and clustering tree of cpKP isolates** **A.** A profile of STs and CGs as well as singletons of our 420 cpKP isolates. These 420 isolates consisted of 313 CG258 ones and 107 non-CG258 ones. The six novel STs identified in this study were highlighted in red. **B.** A maximum-likelihood clustering tree based on the 69,880 core SNPs of the 420 cpKP isolates. *K. variicola* isolate DSM 15968 was used as the outgroup but not shown in the tree. The outer and inner circles in the tree indicated CGs and STs, respectively. MLST, multi-locus sequence typing; ST, sequencing type; CG, clonal group; SNP, single nucleotide polymorphism.

**Supplementary figure S2:**
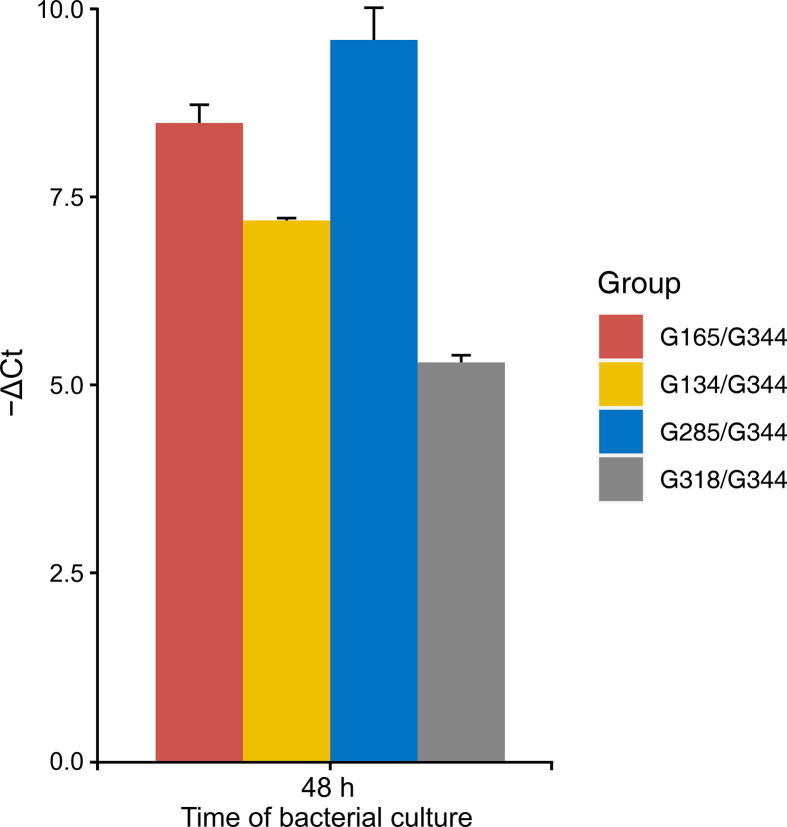
**A population snapshot of the 5099 global KP isolates**. These isolates included our 420 cpKP isolates and the 4679 isolates from the *Klebsiella* MLST database. The lines between STs indicated single-locus variants. The size of circle represented number of isolates. The circles with green rings represented the eight novel STs found in our 420 cpKP isolates, while those with cyan rings stood for the STs found in both our 420 cpKP isolates and the MLST database.

We first performed multi-locus sequence typing (MLST; Figure 2A, Figure S2): 420 cpKP isolates were assigned to 48 STs (including six novel ones: ST3333, ST3334, ST3345, ST3348, ST3349, and ST3350), followed by further assignment to 31 clonal groups (CGs). Among the 420 cpKP isolates, CG258 was the most prevalent CG (313/420, 74.52%), and was composed of four STs: ST11 (298/313, 95.21%), ST2667 (7/313, 2.24%), ST3348 (6/313, 1.92%), and ST258 (2/313, 0.64%). These findings confirm that CG258 accounts for the majority of cpKP isolates, and ST11 is the overwhelmingly dominant ST of CG258 in China.

Next, we performed core-genome clustering analysis of 420 cpKP isolates (Figure 2B). We found that 313 CG258 isolates had gathered at the farthest position from the root, suggesting that CG258 was the latest differentiated clone among the STs/CGs of cpKP isolates. In contrast, 107 non-CG258 isolates were located at earlier splitting branches in the tree. The isolates showed a highly dispersed pattern, illustrating an overall non-clonal population structure with a high level of genetic diversity.

### Phylogeny and evolutionary history of CG258

**Figure 3:**
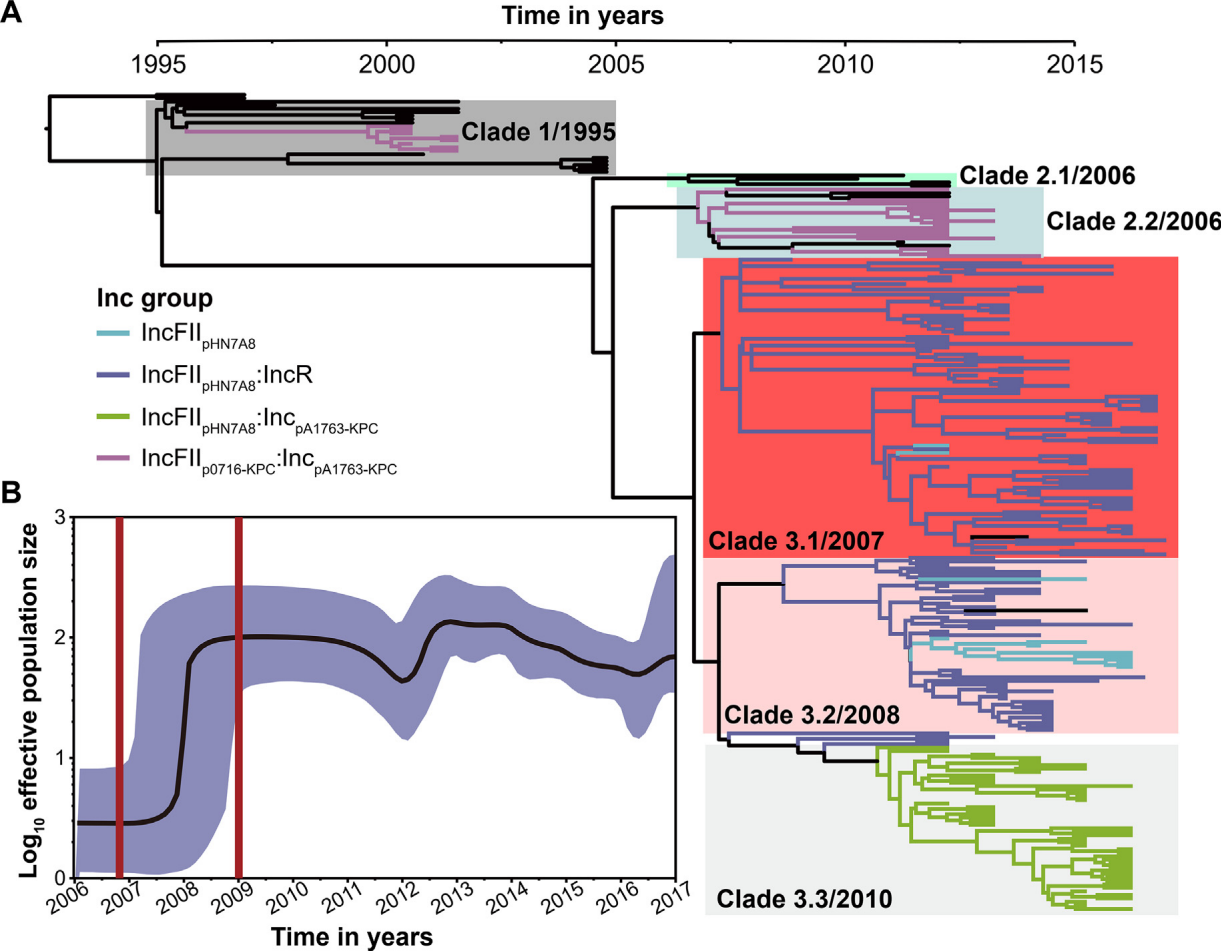
**Evolutionary history of CG258 cpKP isolates** **A.** A time-calibrated MCC Bayesian phylogeny based on the 1271 recombination-free core SNPs. These SNPs came from our 233 non-redundant CG258 cpKP isolates that were shrunk from the total collection of 313 CG258 cpKP ones. The isolates carrying different Inc groups of *bla*_KPC_-carrying plasmids were denoted as distinct colored clusters in the tree. **B.** A Bayesian skyline of the effective population size of the 233 CG258 cpKP isolates. Shadow region indicates 95% probability density interval of estimated population size. MCC, maximum clade credibility; Inc, incompatibility.

We further performed phylogenetic analysis on the CG258 cpKP isolates. A total of 233 non-redundant CG258 cpKP isolates (225 of them belonging to ST11) with 1271 recombination-free single nucleotide polymorphisms (SNPs) were generated. We then constructed a time-calibrated Bayesian maximum clade credibility (MCC) tree for the CG258 cpKP isolates based on the 1271 SNPs (Figure 3A). The results showed that the CG258 cpKP isolates could be divided into three major clades, among which Clade 2 could be further discriminated into two subclades, 2.1 and 2.2, and Clade 3 could be further classified into three subclades: 3.1, 3.2, and 3.3. The emerging time-points of Clades 1, 2.1, 2.2, 3.1, 3.2, and 3.3 were in 1995, 2006, 2006, 2007, 2008, and 2010, respectively.

We further performed a Bayesian skyline plot analysis (Figure 3B), and the results showed a strong population expansion of the CG258 cpKP isolates during 2007–2008, which was consistent with the estimated emergence stage of Clade 3. Notably, Clade 3 was the dominant lineage of the CG258 cpKP isolates, as it accounted for ∼ 80% (186/233) of the CG258 cpKP isolates. Therefore, this population expansion is likely to represent the emergence and subsequent nationwide spread of the dominant Clade 3 of CG258 cpKP isolates in China.

### Prevalence of carbapenemase genes and *bla*_KPC_-carrying plasmids

**Supplementary figure S3:**
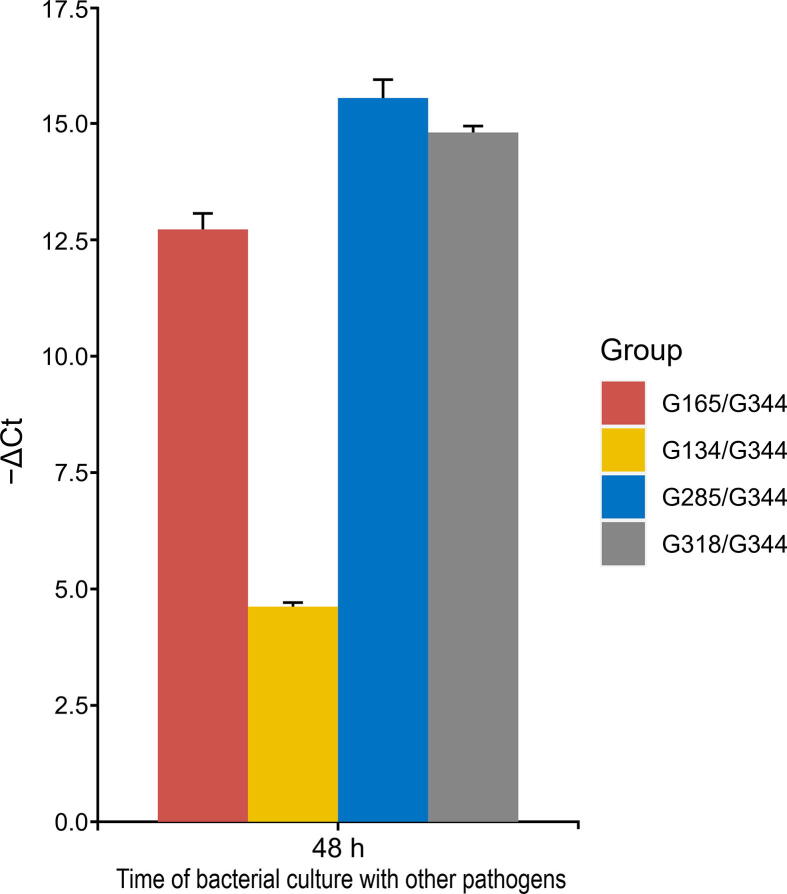
**Prevalence of carbapenemase genes in CG258 and non-CG258 cpKP isolates**. The numbers on the y-axis represented the percentage values. The *P* values were obtained using Fisher exact test. ***, statistically significant with *P*<0.0001.

In the 420 cpKP isolates, we identified three carbapenemase genes: *bla*_KPC-2/-3/-5_ (375/420, 89.29%; in particular, *bla*_KPC-2_ was detected in 372 isolates), *bla*_NDM-1/-5_ (29/420, 6.90%), and *bla*_IMP-4/-38_ (19/420, 4.52%) (Table S3). Most (309/375, 82.43%) of the *bla*_KPC_-carrying isolates belonged to CG258, while the majority of the *bla*_NDM_- and *bla*_IMP_-carrying isolates were assigned into non-CG258 (Figure S3; Table S4). These findings indicated that the dissemination of *bla*_KPC_, as the most prevalent carbapenemase gene, was strongly associated with the spread of CG258 cpKP isolates in China.

**Supplementary figure S4:**
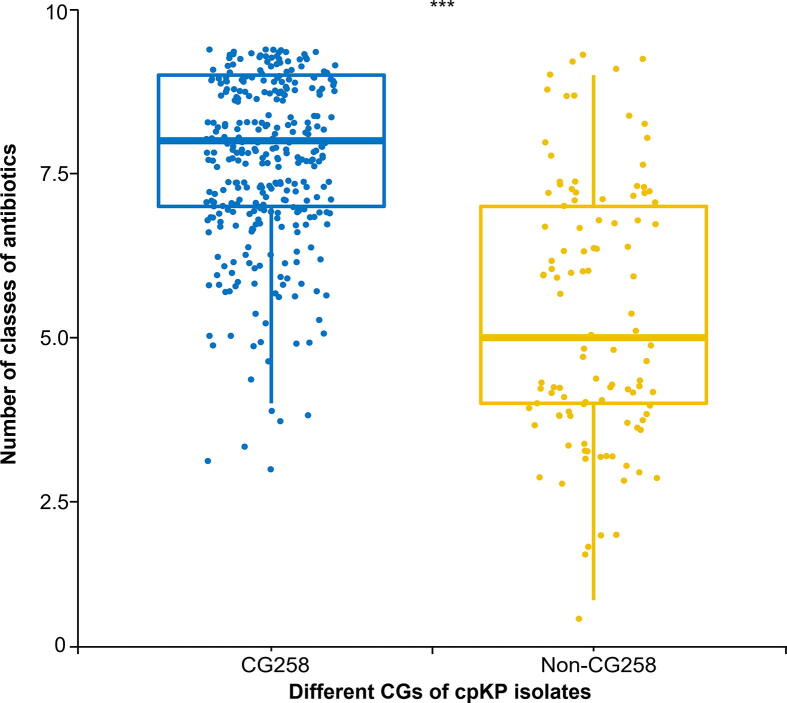
**Alignment of the 28 *bla*_KPC_-carrying IncFII_pHN7A8_ plasmids from our 420 cpKP isolates**. The color rings represented the fully sequenced plasmids, while the grey rings stood for those with draft sequences.

**Supplementary figure S5:**
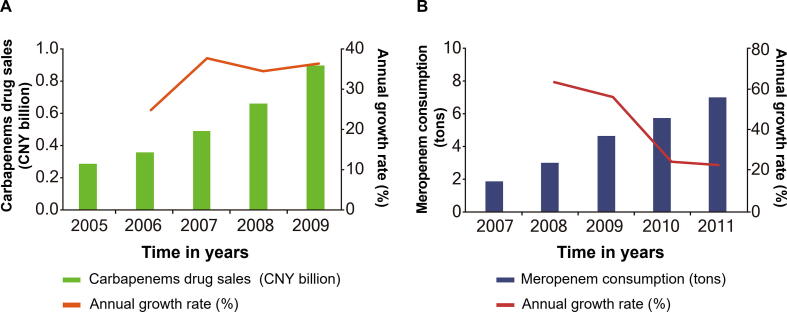
**Alignment of the 163 *bla*_KPC_-carrying IncFII_pHN7A8_:IncR plasmids from our 420 cpKP isolates**. The color rings represented the fully sequenced plasmids, while the grey rings stood for those with draft sequences.

**Supplementary figure S6:**
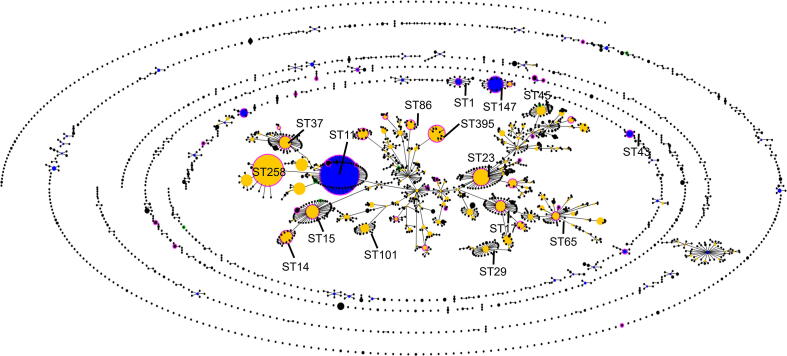
**Alignment of the 59 *bla*_KPC_-carrying IncFII_pHN7A8_:Inc_pA1763-KPC_ plasmids from our 420 cpKP isolates**. The color rings represented the fully sequenced plasmids, while the grey rings stood for those with draft sequences.

**Supplementary figure S7:**
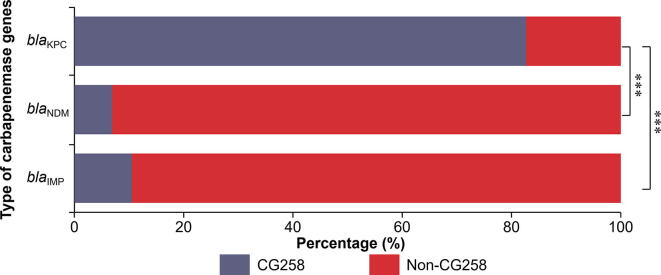
**Alignment of the 30 *bla*_KPC_-carrying IncFII_p0716-KPC_:Inc_pA1763-KPC_ plasmids from our 420 cpKP isolates**. The color rings represented the fully sequenced plasmids, while the grey rings stood for those with draft sequences.

**Supplementary figure S8:**
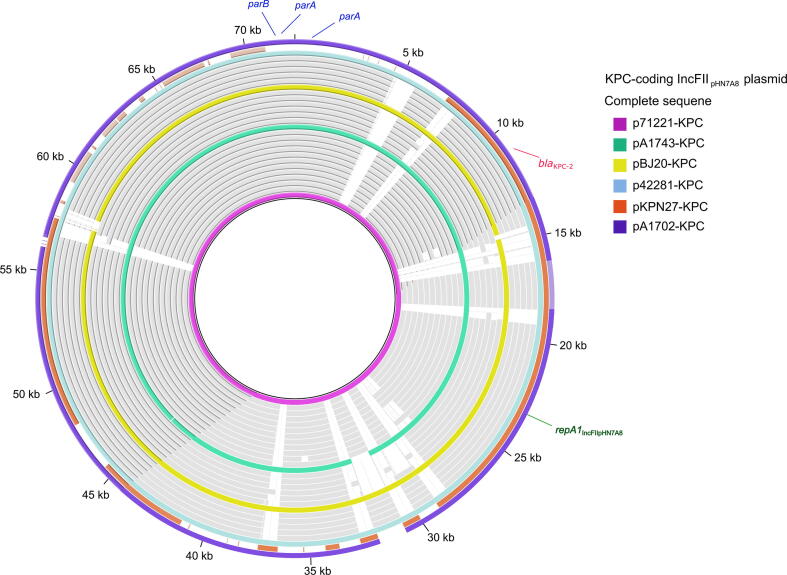
**Alignment of the 36 *bla*_KPC_-carrying IncFII_pKPHS2:_Inc_pA1763-KPC_ plasmids from our 420 cpKP isolates**. The color rings represented the fully sequenced plasmids, while the grey rings stood for those with draft sequences.

All the detected *bla*_KPC_ genes were located in plasmids. A total of 377 *bla*_KPC_-carrying plasmids were identified from the 375 *bla*_KPC_-harboring isolates, with two isolates each carrying a double copy of *bla*_KPC_ in two different plasmids (Table S1). These 377 plasmids could be assigned to 32 Inc groups (Table S5). The top five Inc groups, namely, IncFII_pHN7A8_:IncR (*n* = 163), IncFII_pHN7A8_:Inc_pA1763-KPC_ (*n* = 59), IncFII_pKPHS2_:Inc_pA1763-KPC_ (*n* = 36), IncFII_p0716-KPC_:Inc_pA1763-KPC_ (*n* = 30), and IncFII_pHN7A8_ (*n* = 28), accounted for 83.82% of all *bla*_KPC_-harboring plasmids (Table S5). The plasmids of each Inc group carried identical core backbone *rep* and *par* genes but showed considerable modular divergence across their whole genomes (Figures S4–S8). Overall, although the *bla*_KPC_-harboring plasmids in cpKP isolates owned the diversity of Inc groups (Table S5), the aforementioned five main Inc groups were dominant.

### Correlation between three major Inc groups of *bla*_KPC_-carrying plasmids and CG258

**Figure 4:**
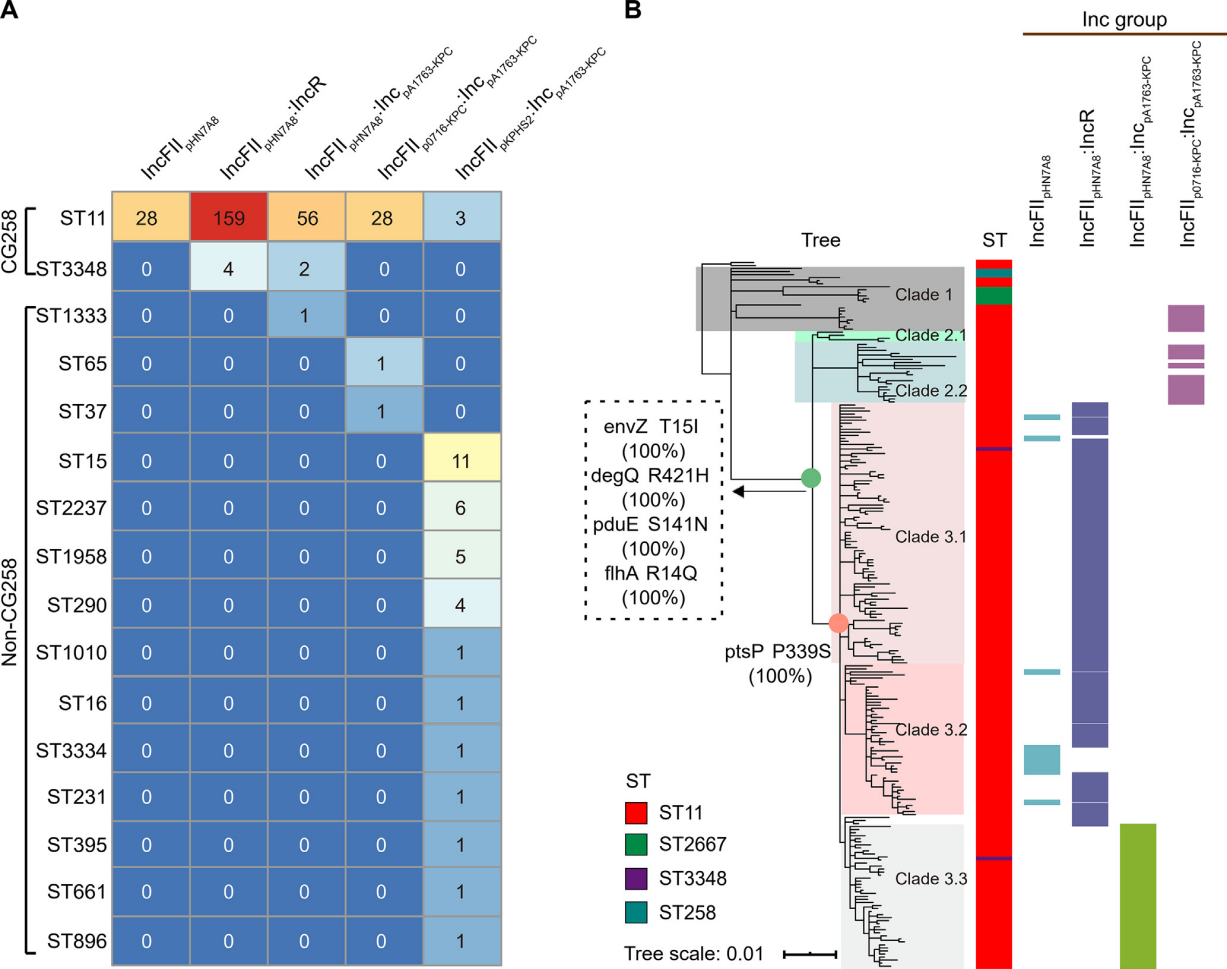
**Correlation of different Inc groups of *bla*_KPC_-carrying plasmids with CG258 and non-CG258** **A.** Prevalence of the top five Inc groups (Table S5) among our CG258 and non-CG258 cpKP isolates. The numbers in the cells represent the numbers of cpKP isolates (316 in total). **B.** Association of the four Inc groups with different clades of CG258. The tree was the MCC Bayesian tree of the 233 CG258 cpKP isolates. The SNPs on the nodes indicated some specific markers for the accurate classification of Clades 1, 2, and 3 of ST11/CG258 cpKP isolates.

We further analyzed the relationships between the five major Inc groups of *bla*_KPC_-harboring plasmids and the corresponding cpKP isolates. The results showed that three major Inc groups of *bla*_KPC_-carrying plasmids (IncFII_pHN7A8_, IncFII_pHN7A8_:IncR, and IncFII_pHN7A8_:Inc_pA1763-KPC_) had a strong correlation with the CG258 cpKP isolates (Figure 4).

On the one hand, almost all the three Inc groups of *bla*_KPC_-carrying plasmids (249/250, 99.60%) were detected in the CG258 cpKP isolates, which occupied a very large proportion of the CG258 cpKP isolates (249/313) (Figure 4A). In addition, we downloaded all the 38 complete genomes of ST11/CG258 cpKP isolates from NCBI, and 77.5% of them harbored these three Inc groups of plasmids (Table S6). Previous studies have also demonstrated the strong association between ST11/CG258 cpKP isolates and the three IncFII-like plasmids [6], [18].

We next mapped the Inc groups of *bla*_KPC_-carrying plasmids onto the phylogenetic tree of CG258 cpKP isolates, and observed a strong correlation of IncFII_pHN7A8_ (13/13, 100%) and IncFII_pHN7A8_:IncR (119/122, 97.54%) with Clade 3.1+3.2, and IncFII_pHN7A8_:Inc_pA1763-KPC_ (45/45, 100%) with Clade 3.3 (Figure 4B). Consistently, Clade 3.1+3.2 (132/136, 97.06%) showed a strong correlation with IncFII_pHN7A8_ and IncFII_pHN7A8_:IncR, and Clade 3.3 (45/47, 95.74%) with IncFII_pHN7A8_:Inc_pA1763-KPC_ (Figure 4B). Here, we have referred to them as the three advantageous bacterium–plasmid combinations due to the strong correlation: Clade 3.1+3.2–IncFII_pHN7A8_, Clade 3.1+3.2–IncFII_pHN7A8_:IncR, and Clade 3.3–IncFII_pHN7A8_:Inc_pA1763-KPC_.

### Acquisition of three IncFII_pHN7A8_-related Inc groups of *bla*_KPC_-carrying plasmids promoted nationwide spread of CG258

The three bacterium–plasmid combinations of the Clade 3 CG258 cpKP isolates and the IncFII_pHN7A8_-related Inc groups of *bla*_KPC_-carrying plasmids represented the dominant lineage of CG258 cpKP isolates in China. This implies that these three genotypic combinations have certain phenotypic advantages over the other two main Inc groups.

**Figure 5:**
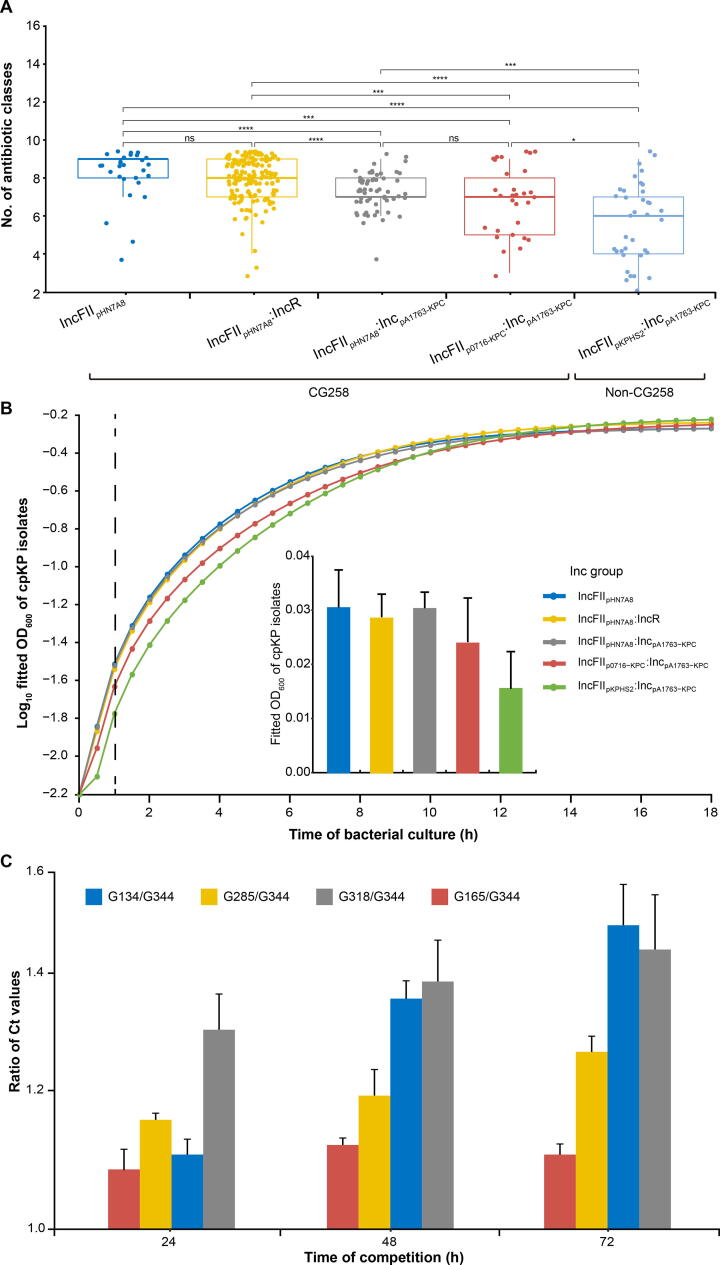
**Resistance, growth, and competition advantages of cpKP harboring *bla*_KPC_-carrying plasmids of different Inc groups** **A.** Boxplot showing the numbers of classes of antibiotics that different subgroups of cpKP isolates were resistant to. The *P* values were obtained using Kruskal–Wallis, a non-parametric test for the comparison of multiple groups. *, *P* < 0.05; **, *P* < 0.01; ***, *P* < 0.001; ****, *P* < 0.0001. **B.** Bacterial growth curves of different subgroups of cpKP isolates. The dashed line indicates the time point at 1 h, when bacteria were at the logarithmic growth phases; corresponding OD_600_ values (mean ± standard error) were shown in the embedded bar plot. **C.** Bacterial *in vitro* competition experiments. Shown were the ratio of Ct values (mean ± standard error) between each two cpKP isolates at 24 h, 48 h, and 72 h, respectively.

**Supplementary figure S9:**
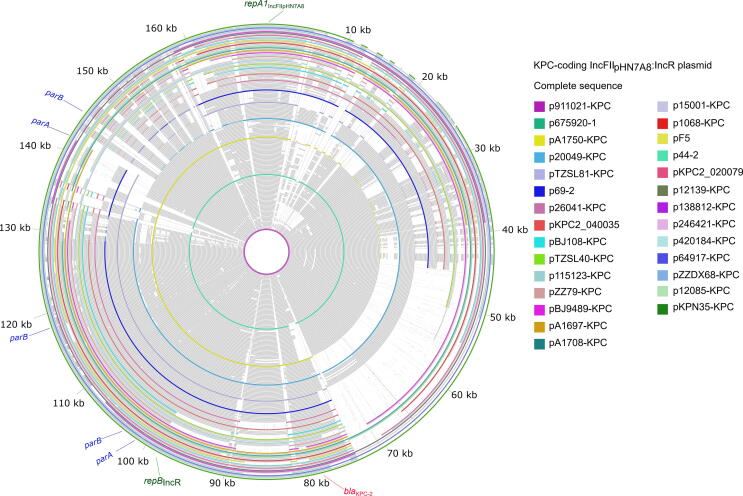
**Antimicrobial susceptibility data of cpKP isolates**. Shown were the drug resistance profile of our 420 cpKP isolates (**A**), and the resistance rates (**B**) (resistant / [sensitivity + resistant]) of CG258 and non-CG258 isolates for each indicated antibiotics. A total of 21 antibiotics in nine classes were tested. The p values were obtained using Fisher’s exact test. NS, no significant difference. ** and ***, statistically significant with *P*<0.001 and *P*<0.0001, respectively.

We performed drug resistance, growth, and competition experiments to validate the phenotypic advantages of these genotypic combinations (Figure 5). We first examined the susceptibility/resistance profiles of the 420 cpKP isolates to 9 classes of 21 different antibiotics (Figure S9; Table S1). In general, the combinations are ranked from the highest to the lowest level of resistance: CG258 isolates harboring three IncFII_pHN7A8_-related Inc groups of *bla*_KPC_-carrying plasmids > CG258 isolates harboring IncFII_p0716-KPC_:Inc_pA1763-KPC_ of *bla*_KPC_-carrying plasmids > non-CG258 isolates harboring IncFII_pKPHS2_:Inc_pA1763-KPC_ of *bla*_KPC_-carrying plasmids (Figure 5A). A similar trend was also observed for the bacterial growth rates under antibiotic treatment (Figure 5B).

The *in vitro* competitive assay was then carried out using five representative *bla*_KPC_-carrying cpKP isolates of G134, G285, G318, G165, and G344. Each of the five cpKP isolates contains a single plasmid: G134 (ST11 + IncFII_pHN7A8_), G285 (ST11 + IncFII_pHN7A8_:IncR), G318 (ST11 + IncFII_pHN7A8_:Inc_pA1763-KPC_), G165 (ST11 + IncFII_p0716-KPC_:Inc_pA1763-KPC_), and G344 (non-CG258 + IncFII_pKPHS2_:Inc_pA1763-KPC_). The results showed a similar trend: isolates harboring three IncFII_pHN7A8_-related Inc groups > isolates harboring IncFII_p0716-KPC_:Inc_pA1763-KPC_ > isolates harboring IncFII_pKPHS2_:Inc_pA1763-KPC_ (Figure 5C).

**Supplementary figure S10:**
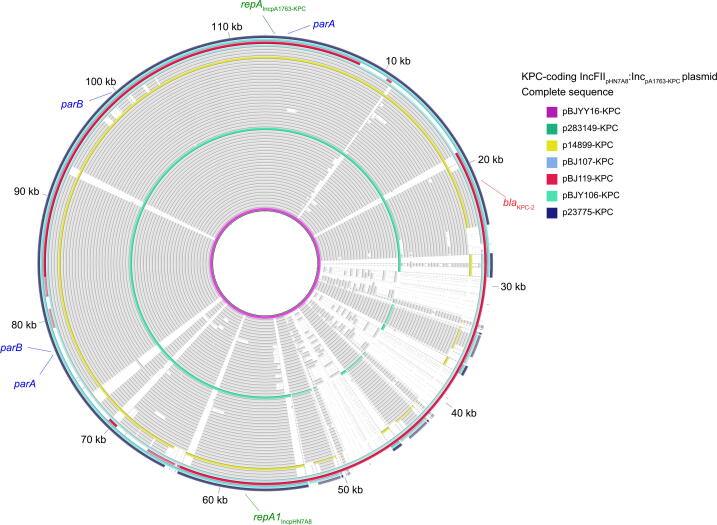
***In vitro* competition experiments of four pairs of cpKP isolates in supernatants**. The -ΔCt values (mean ± standard error; -ΔCt value = CtG344 - Ct_G134/G285/G318/G165_ values) for the four pairs of cpKP isolates (G134/G344, G285/G344, G318/G344, and G165/G344) were determined at 48 h.

The competitive assay was further performed by using the supernatants of four mixtures (G134 & G344, G285 & G344, G318 & G344, and G165 & G344) at 48 h of culture. The results showed higher competitive growth capacities in the four ST11/CG258 isolates (G134, G285, G318, and G165) over non-CG258 isolate (G344) (Figure S10), which was consistent with the results of genomic DNA analysis of the bacterial precipitates (Figure 5C).

**Supplementary figure S11:**
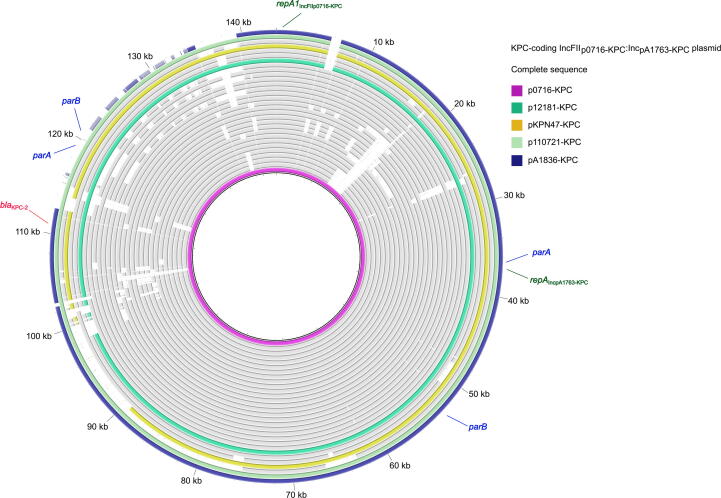
***In vitro* competition experiments under co-cultivation with other three carbapenemase-producing pathogens**. The -ΔCt values (mean ± standard error; -ΔCt value = Ct_G344_ - Ct_G134/G285/G318/G165_ values) between each pair of cpKP isolates (G134/G344, G285/G344, G318/G344, and G165/G344) were measured at 48 h.

To determine whether the findings from the competitive assays were affected by other pathogens, three carbapenemase-producing pathogens (*Escherichia coli* 14406, *Pseudomonas aeruginosa* SE5419, and *Acinetobacter baumannii* HBYA24) were selected for co-culture with four mixtures (G134 & G344, G285 & G344, G318 & G344, and G165 & G344) which are known as the co-infection pathogens with *K. pneumoniae*. Consistently, these four ST11/CG258 isolates (G134, G285, G318, and G165) appeared to have an advantage in terms of growth over the non-CG258 isolate (G344) at 48 h of co-culture with the other pathogens (Figure S11).

The findings of the aforementioned assays indicate that the acquisition of the three IncFII_pHN7A8_-related Inc groups of *bla*_KPC_-carrying plasmids rendered their host *K. pneumoniae* the higher levels of antibiotic resistance and competitive advantageous growth. These phenotypic advantages might promote the nationwide spread of the dominant Clade 3 of CG258 cpKP isolates in China.

### Optimized antibiotic combination regimens for treatment of infection with cpKP

**Supplementary figure S12:**
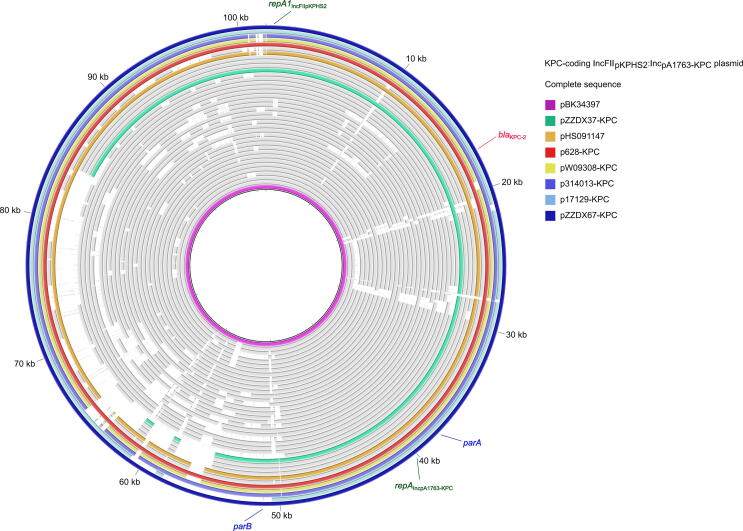
**Boxplots showing the numbers of classes of antibiotics that CG258 and non-CG258 isolates were resistant to**. The *P* value was tested using Wilcoxon test. ***, statistically significant with *P*<0.0001.

All the 420 cpKP isolates were found to be resistant to β-lactams, including carbapenems, whereas the resistant rates of these isolates against aminoglycosides [amikacin (47.86%, 201/420), tobramycin (57.62%, 242/420), and gentamicin (79.52%, 334/420)] and trimethoprim/sulfamethoxazole (65%, 273/420) were much lower (Figure S9A). In addition, the CG258 isolates displayed much stronger drug resistance levels than the non-CG258 isolates both in terms of the drug resistance rate and the antibiotic classes (Figures S9B and S12). Specifically, CG258 isolates exhibited the lowest resistance against amikacin (57.4%, 179/312) followed by trimethoprim/sulfamethoxazole (67.7%, 212/313) and tobramycin (73.2%, 202/276), while non-CG258 isolates exhibited the lowest resistance against amikacin (20.6%, 22/107) followed by tobramycin (50.6%, 40/79) and levofloxacin (53.9%, 55/102) (Figure S9B). These differences in the resistance profiles between CG258 and non-CG258 isolates led us to investigate which antibiotic combination regimens are optimal for the treatment of infection with CG258 and non-CG258 cpKP.

**Figure 6:**
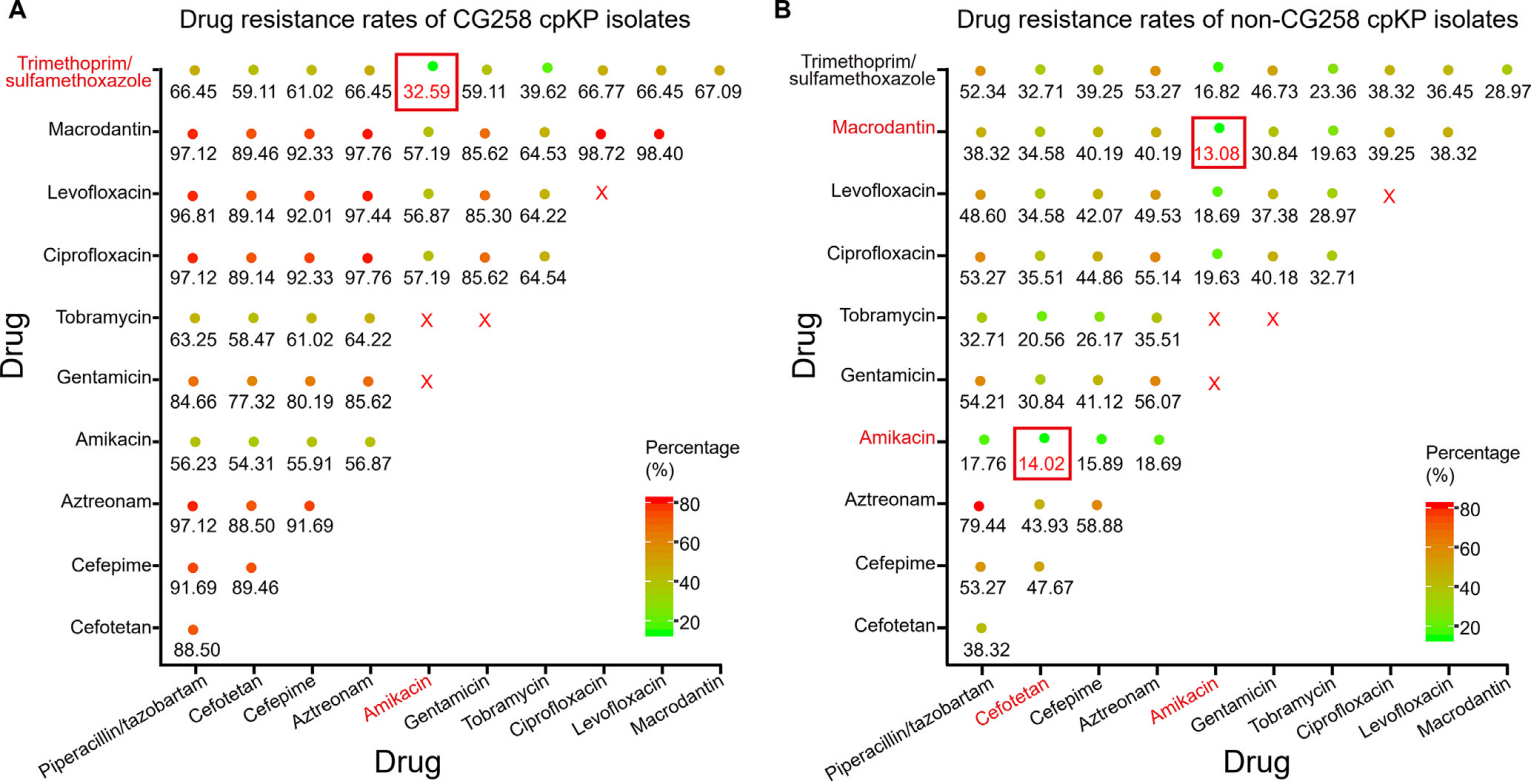
**Optimized two-antibioticcombination regimens for treatment of CG258 and non-CG258** Shown were the resistance rates (the numbers of isolates resistant to both antibiotics tested/the total numbers of isolates) when different two-antibiotic combination regimens were used for the treatment of CG258 (**A**) and non-CG258 (**B**). The drug resistance rate of each two different drug combinations of 11 antibiotics (excluding 10 antibiotics with the resistance ratio of ∼ 100% and some banned drug combinations) was calculated. The size of the solid circles increases with the increase in resistance rate. The red rectangle indicates the optimized drug combinations of two drugs. The red cross indicates the prohibited drug combinations since they belong to the same type of antimicrobial drug.

Based on the calculated resistance ratios when different two-antibiotic combination regimens were used for CG258 treatment, two optimal combinations of ‘amikacin + trimethoprim/sulfamethoxazole’ and ‘tobramycin + trimethoprim/sulfamethoxazole’ produced the resistance ratios of 32.59% (102/313) and 39.62% (124/313), respectively, which were much lower than the ratio of 57.4% (179/312) calculated for optimal single antibiotic ‘amikacin’ (Figure 6A). In addition, the ‘amikacin + macrodantin’ and ‘amikacin + cefotetan’ combinations represented the two optimal two-antibiotic combinations against non-CG258 isolates, with the resistance rates of 13.08% (14/107) and 14.02% (15/107), respectively (Figure 6B).

## Discussion

**Figure 7:**
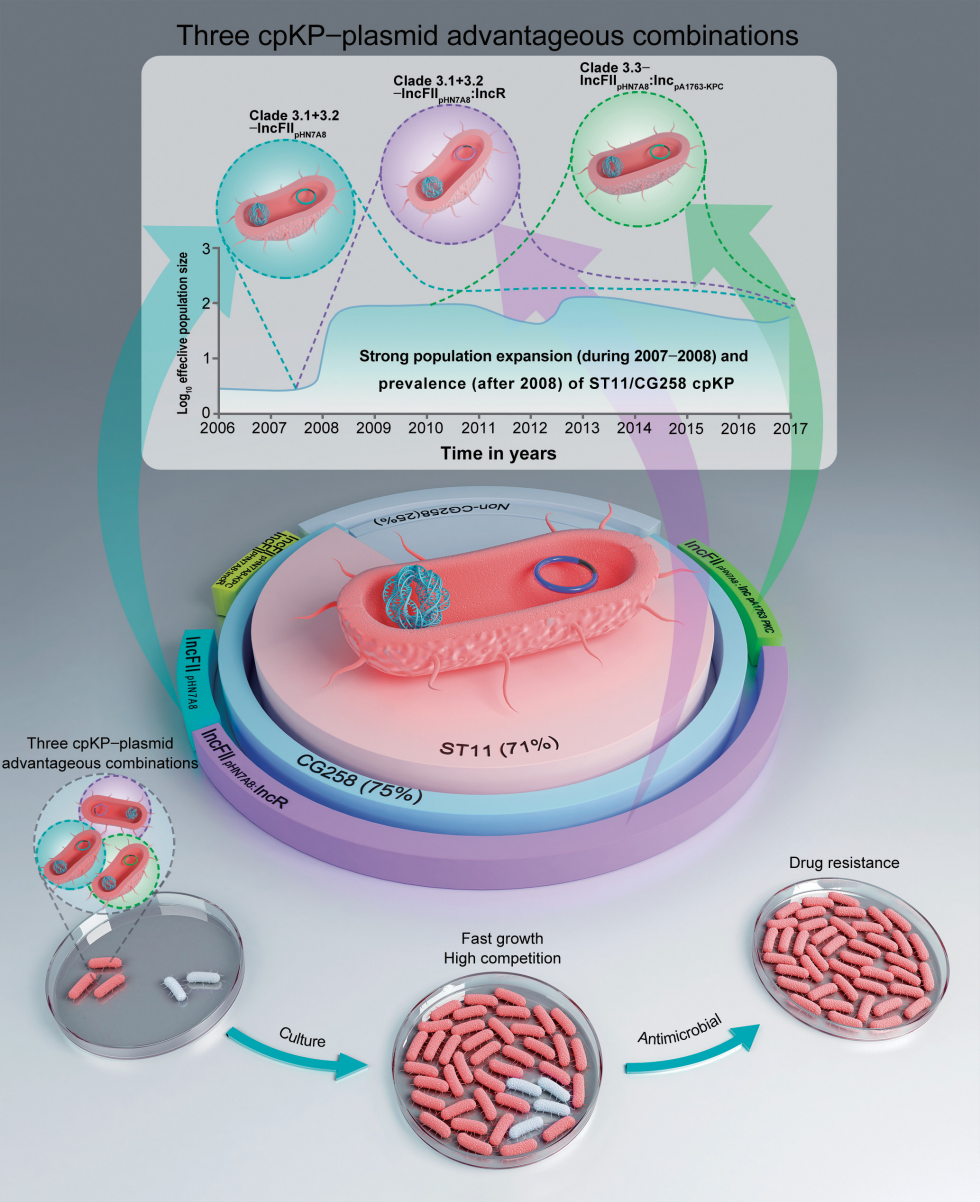
**Three advantageous host–plasmid combinations** The three advantageous host–plasmid combinations (Clade 3.1+3.2–IncFII_pHN7A8_, Clade 3.1+3.2–IncFII_pHN7A8_:IncR, and Clade 3.3–IncFII_pHN7A8_:Inc_pA1763-KPC_) that led to the strong population expansion during 2007–2008 and subsequent maintenance of the prevalence and clonal dissemination of ST11/CG258 cpKP isolates after 2008.

Our findings provide strong evidence that the three host–plasmid advantageous combinations identified here play important and even decisive roles in the successful clonal spread of ST11/CG258 cpKP in China. These three advantageous combinations represent the main phylogenetic subclades of ST11/CG258 cpKP isolates that carry the most prevalent Inc groups of KPC-producing plasmids: Clade 3.1+3.2–IncFII_pHN7A8_, Clade 3.1+3.2–IncFII_pHN7A8_:IncR, and Clade 3.3–IncFII_pHN7A8_:Inc_pA1763-KPC_ (Figure 2, Figure 3B and Figure 2, Figure 3B). The advantages of these isolates were reflected in both genotypic and phenotypic aspects with features of strong genomic correlation and coevolution in host–plasmid, and highly related drug resistance, growth, and competition in phenotypes. Moreover, both the genomic and phenotypic advantages exhibited reciprocal causation, which indicates high adaptability of these three IncFII_pHN7A8_-related plasmids to ST11/CG258 cpKP isolates, and explains their successful clonal dissemination in China (Figure 7).

### Three advantageous combinations of host KP**–**plasmid as the major genetic basis for the nationwide spread of ST11/CG258 cpKP

Our results showed that acquisition of the three IncFII_pHN7A8_-related *bla*_KPC_-carrying plasmids, especially the IncFII_pHN7A8_:IncR and IncFII_pHN7A8_:Inc_pA1763-KPC_ plasmids, endow the ST11/CG258 isolates with the phenotypic advantages of having a greater antibiotic resistance and higher competitive growth rate. Previous studies have also reported the clonal dissemination of CG258 cpKP and demonstrated the important roles of the IncFII *bla*_KPC_-carrying plasmids in CG258 cpKP isolate related drug resistance in China and European countries [6], [18]. Our study further distinguished the roles of different IncFII groups of *bla*_KPC_-carrying plasmids, and we found that the IncFII_pHN7A8_:IncR and IncFII_pHN7A8_:Inc_pA1763-KPC_ plasmids were high risk. These findings improve our understanding of the genetic basis for the nationwide spread of ST11/CG258 cpKP.

IncFII_pHN7A8_:IncR and IncFII_pHN7A8_:Inc_pA1763-KPC_ plasmids are chimeras of IncFII_pHN7A8_ and IncR/Inc_pA1763-KPC_ plasmids [19], [20], [21], [22]. Compared to single-replicon plasmids, multi-replicon plasmids have the advantages of rapid replication, replicon substitution, multiple partitioning and/or toxin-antitoxin systems for plasmid maintenance, and a high survival rate. In particular, these two chimeric plasmids possess the following additional advantages. First, the IncR or Inc_pA1763-KPC_ backbones are very small, which lowers the cost of adaption of the chimeras after their fusion with IncFII_pHN7A8_. Second, IncR and Inc_pA1763-KPC_ plasmids carry large multidrug resistance regions, thus expanding the resistance profile of their hosts. In addition, although IncR and Inc_pA1763-KPC_ plasmids do not carry conjugation transfer regions, they can acquire self-transfer ability after fusion with conjugative IncFII_pHN7A8_ plasmids, further facilitating the spread of the resistance genes they carry.

### Widespread use of carbapenems as the leading external cause for the expansion and prevalence of ST11/CG258 cpKP

**Supplementary figure S13:**
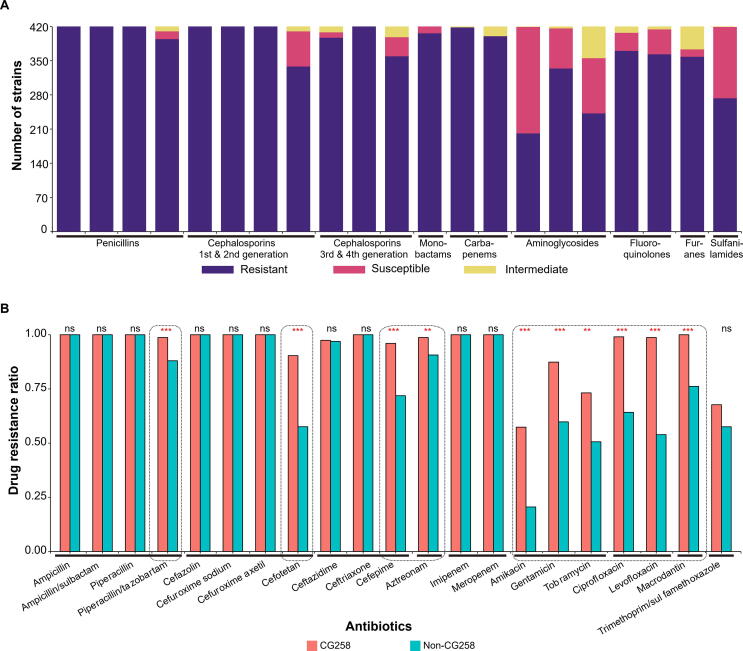
**Carbapenem sales/consumption in China**. **A**. Carbapenem sales in China from 2005 to 2009. **B**. Meropenem consumption in China from 2007 to 2011.

There has been a rapid increase in the sale and use of carbapenems since 2005, and the highest growth rate was observed during 2007–2008 (Figure S13) [23]. This is the major external factor that has driven antibiotic resistance, clonal expansion, and consequently the nationwide spread of ST11/CG258 cpKP in China.

Specifically, the consumption of meropenem increased sharply from 2007 to 2009 (Figure S13) [23]. Meanwhile, some other carbapenems were listed in China and facilitated their consumption (ertapenem in 2005, faropenem in 2006, biapenem in 2008). Therefore, the rapid growth of carbapenem consumption around 2007 might have directly led to the expansion of ST11/CG258 cpKP. After 2008, this trend plateaued out as a result of the slow growth of sales of carbapenems in China [24], [25]. Additionally, restrictions on antibiotic prescription by the Ministry of Health of China since 2011 have also resulted in a significant decrease in the use of antibiotics in many hospitals across the country [24], [25]. Further, the Chinese government has introduced several policies to strictly control the usage of carbapenem antibiotics across 1429 hospitals (https://www.carss.cn/).

In conclusion, both internal (three host–plasmid advantageous combinations) and external (widespread use of carbapenems) factors may have together facilitated the spread and prevalence of ST11/CG258 cpKP in China. Therefore, close monitoring of host–plasmid combinations and more strict policies on the prescription and sale of antibiotics are needed to effectively inhibit the epidemic spread of cpKP.

### Application of precision medicine for the treatment of cpKP infections by monitoring the adaptability of resistant plasmids

In the present study, the cpKP isolates displayed the lowest resistance rates for three aminoglycosides (amikacin, tobramycin, and gentamicin) and trimethoprim/sulfamethoxazole, which was consistent with many other published studies (Table S7). Additionally, our findings revealed that CG258 cpKP isolates had broader resistance profiles than non-CG258 cpKP isolates (Figure S12), which is supported by the epidemiological survey data on carbapenem-resistant *Enterobacteriaceae* from 2012 to 2016 in China [16], [26]. These findings provide the possibility of optimizing two-antibiotic combination regimens for the treatment of CG258/non-CG258 infections by calculating their resistance rates in response to different antibiotic combinations. However, more in-depth studies on different antibiotic combination regimens need to be conducted using various cpKP isolates with different Inc groups of plasmids, in order to provide precise references for the effective treatment of cpKP infection with existing drugs. Further studies on such therapeutic combinations would be clinically valuable and worthy of investigation in the clinical setting.

Eventually, we hope that our study helps health authorities closely monitor the adaptability of resistant plasmids, so as to effectively prevent the emergence and dissemination of new advantageous host–plasmid combinations. Additionally, we should consider both genotypes and Inc groups of drug-resistant plasmids for achieving precision medicine for cpKP infections.

## Conclusion

In summary, our study systematically demonstrated the molecular epidemiology and genetic basis for the dissemination of ST11/CG258 cpKP in China. We conducted a comprehensive genomic epidemiology analysis of 420 cpKP isolates from 70 hospitals in 24 Chinese provinces/autonomous regions/municipalities during 2009–2017 based on short-/long-read sequencing. We found that three advantageous combinations of host–*bla*_KPC_-carrying plasmids (Clade 3.1+3.2–IncFII_pHN7A8_, Clade 3.1+3.2–IncFII_pHN7A8_:IncR, and Clade 3.3–IncFII_pHN7A8_:Inc_pA1763-KPC_) endowed the cpKP isolates with advantageous genotypic (strong correlation and coevolution between the bacterium and the plasmids) and phenotypic (high rates of resistance, growth, and competition) characteristics, thereby facilitating the nationwide spread of ST11/CG258 cpKP. Intriguingly, Bayesian skyline plot analysis illustrated that the three advantageous combinations might be directly associated with the strong population expansion during 2007–2008 and subsequent maintenance of the ST11/CG258 cpKP population after 2008. We then assessed the drug resistance profiles and proposed combination treatment regimens for CG258/non-CG258 cpKP. In particular, our findings indicate that the Inc group of drug-resistant plasmids should be considered when developing precision medicine for cpKP infections, and the advantageous cpKP–plasmid combinations should be monitored closely to prevent the dissemination of ST11/CG258 cpKP isolates in China.

## Materials and methods

### Bacterial isolates and genomic DNA extraction

We collected 2803 clinical KP isolates from 70 hospitals in 24 provinces/autonomous regions/municipalities of China from 2009 to 2017. After eliminating 59 unsuccessfully cultured isolates, 2744 isolates were obtained for PCR detection of KP-specific *khe* gene to identify the species. After excluding the 53 isolates without *khe* gene, 2691 isoates were further tested to produce carbapenemases by Modified Carba NP test. The result showed that 493 isolates were confirmed to produce carbapenemases. Bacterial genomic DNA was then extracted using a Qiagen UltraClean Microbial DNA Isolation Kit (Catalog No. 12224-50, Qiagen, Germany), and then sequenced by Illumina technology. After excluding 73 low-quality sequencing samples, 420 cpKP genomes were used for the subsequent analysis (Figure 1; Table S1).

### Genome sequencing and assembly

The draft genome sequences of bacterial genomic DNA were sequenced from a paired-end library with an average insert size of 350 bp on an Illumina HiSeq2000 sequencing platform [27]. The sequencing libraries for Illumina were constructed using the NEB Next Ultra DNA Library Preparation Kit (Catalog No. E7370L, New England Biolabs, MA) according to the library preparation workflow of the manufacturer’s recommendation [25]. Adapters and low-quality reads were removed using FASTX-Toolkit (https://hannonlab.cshl.edu/fastx_toolkit/). SPAdes v3.9.0 [28] was used to do the *de novo* assembly from the trimmed sequence reads using *k*-mer sizes of 21, 33, 55 and the -cov-cutoff flag set to ‘auto’. Isolates were discarded through the following criteria. First, if the size of the *de novo* assembly was outside of 5–7 Mb, isolates were discarded. Second, if the average nucleotide identity to NJST258_1 was lower than 95% or the top match was not NJST258_1 after the *de novo* assembly was compared with the reference genomes of five *Klebsiella* spp. (*K. pneumoniae* NJST258_1, CP006923; *K. quasipneumoniae* ATCC 700603, CP014696; *K. michiganensis* E718, NC_018106; *K. oxytoca* CAV1374, CP011636; *K. variicola* DSM 15968, CP010523) using Pyani-0.2.7 [29], the isolates were discarded. In addition, if the percentage of the total number of genomic sites with more than 10-fold depth of coverage was lower than 80% after the raw sequencing reads of each isolate were mapped to the NJST258_1 genome using Bowtie2 [30], and the depth for each position on the genome was calculated using SAMtools depth v0.1.19 [31], the isolates were also discarded.

The complete genome sequences were obtained from a sheared DNA library with an average size of 10 kb on a PacBio RSII sequencer (Pacific Biosciences) [32], [33]. The library was prepared using sheared genomic DNA (> 5 µg) through the PacBio 10-kb SMRT-bell Template Preparation Kit (Pacific Biosciences, CA) according to the library preparation workflow of the manufacturer’s recommendation [32], [33]. And then the *de novo* sequence assembly was performed using SMRT Analysis v2.3.0 (https://smrt-analysis.readthedocs.io/en/latest/). Nanopore GridION platform was also used for whole-genome sequencing [34]. Here, the sequencing libraries were prepared using 3 μg of purified DNA through the Ligation Sequencing Kit (SQK-LSK109, Oxford Nanopore Technologies, UK) [34]. The high-quality reads (mean_qscore_template ≥ 7 and length ≥ 1,000) were screened for further *de novo* sequence assembly using Canu (https://canu.readthedocs.io/en/latest/) [35]. Circularization of chromosomal or plasmid sequences was achieved by manual comparison. Pilon v.1.13 [36] was employed to polish complete genome sequences using Illumina sequencing reads.

The accession numbers for the reference plasmids are listed in Table S8.

### MLST

SRST2 [37] was used to identify the ST of each KP isolate by mapping its Illumina sequencing reads to the Pasteur *Klebsiella* MLST Database (https://bigsdb.pasteur.fr/klebsiella/klebsiella.html). All the STs in the *Klebsiella* MLST database (last accessed August 3, 2018) were assigned to different CGs using *eBURST* [38].

### Construction of recombination-free Bayesian phylogenetic tree

Our 313 CG258 cpKP isolates were subjected to sequence alignment. Recombination DNA regions were predicted using ClonalFrameML [39], followed by removal of all putative recombinant SNP sites (r/m = 3.41). A Bayesian phylogenetic tree was constructed from the recombination-free core SNPs of the 233 non-redundant CG258 cpKP isolates using MrBayes [40] and visualized using iTOL (https://itol.embl.de/).

### Bayesian phylogenetic inference and molecular dating analyses

Bayesian skyline analysis was performed to calculate the change in the effective population size of the aforementioned 233 isolates using BEAST v1.8.4 [41]. The three standard substitution models, Hasegawa-Kishino-Yano (HKY), general time-reversible (GTR), and Tamura-Nei 93 (TN93) were tested in combination with the estimated/empirical base frequency, the gamma (G) site heterogeneity, and the loose molecular clock. By testing various parameter combinations, the model combination “GTR + empirical + G4” was selected. The tip date was defined as the sampling time. In the end, three independent chains of 5 × 10^7^ generations were run to ensure calculation accuracy, with sampling every 1000 iterations. The resulting Bayesian skyline plot was visualized using Tracer v1.7 [42]. A time-calibrated Bayesian MCC tree of the aforementioned 233 isolates was constructed using TreeAnnotator (https://beast.community/treeannotator) and visualized using FigTree (https://tree.bio.ed.ac.uk/software/figtree/).

### Plasmid analysis

All the fully sequenced *bla*_KPC_-carrying plasmids from GenBank (last accessed Aug 29, 2018) and our study were used as references. The draft sequences of the rest *bla*_KPC_-carrying plasmids in our 420 cpKP isolates were aligned using BLAST [43] and custom Perl scripts. Inc groups and core backbone *rep* and *par* genes were determined for all the *bla*_KPC_-carrying plasmids in our 420 cpKP isolates. To ensure accuracy, the assembled draft plasmid sequences met the following three criteria [44]. First, the *bla*_KPC_-embedded contigs had 100% query coverage and ≥ 99% identity with corresponding reference plasmids. Second, the *bla*_KPC_-embedded contigs and the *rep*-embedded contigs of the same plasmid had similar sequencing depth. In addition, each draft plasmid sequence had ≥ 70% query coverage and ≥ 94% identity with corresponding reference plasmids.

### Identification of carbapenemase genes and virulence genes

The major plasmid-borne carbapenemase genes were screened for each cpKP isolate by PCR, followed by amplicon sequencing using ABI 3730 Sequencer [45]. The variants of *bla*_KPC_, *bla*_NDM_, and *bla*_IMP_ were identified from genome sequence data using ResFinder [46]. The virulence genes were identified using BLAST based on the database from Pasteur Institute (https://bigsdb.web.pasteur.fr/klebsiella/klebsiella.html) (Table S2).

### Bacterial phenotypic resistance assays

Bacterial antimicrobial susceptibility was tested by the BioMérieux VITEK 2 AST-GN09 TEST KIT (Catalog No. 22008, BIOMERIEUX, France) and interpreted based on the 2018 Clinical and Laboratory Standards Institute (CLSI) guidelines [47]. The activity of Ambler class A/B/D carbapenemases in bacterial cell extracts was determined by a modified CarbaNP test [45].

### Bacterial growth curves

Bacterial growth curves of the 420 cpKP isolates were measured on a 96-well microtiter plate using a Thermo Scientific Multiskan FC instrument. The equivalent amount of overnight bacterial culture was added in each well containing 200 μl of LB liquid medium (4 mg/l meropenem), and the mixtures were cultured at 37 °C overnight with a speed of 5 Hz. Negative controls (200 μl bacteria-free LB liquid medium) were also included in the study. The bacterial growth curve was determined through a course of time by recording the turbidity at 600 nm using the microplate reader of the Multiskan FC instrument. Experiments were performed in triplicate.

### *In vitro* competition experiments

An equivalent amount of overnight bacterial cultures of two indicated bacterial isolates were inoculated into 10 ml of LB liquid medium (4 mg/l meropenem), and the mixtures were cultured at 37 °C for 72 h in a shaker with a speed of 200 r/min. At 0 h, 24 h, 48 h, and 72 h, 3 ml aliquots of the cultures were taken, and genomic DNA was extracted. To examine the competition between two bacterial isolates, we performed real-time qPCR to determine the ratio of Ct values between each of the four ST11/CG258 isolates (G134, G285, G318, and G165) and the control non-CG258 isolate G344. The five specific genes on chromosome (G134_05212, G285_01367, G318_02254, G165_02217, and G344_00764) were selected as PCR target sequences, and the corresponding PCR primers are listed in Table S9. Experiments were performed in triplicate.

## Code availability

The code for phylogenetic analyses for *Klebsiella pneumoniae* has been submitted to BioCode at the National Genomics Data Center (NGDC), Beijing Institute of Genomics (BIG), Chinese Academy of Sciences (CAS) / China National Center for Bioinformation (CNCB), and are publicly accessible at https://ngdc.cncb.ac.cn/biocode/tools/7276.

## Data availability

The genome sequences in this study have been deposited in the Genome Sequence Archive [48] at the NGDC, BIG, CAS / CNCB (GSA: CRA003059), and are publicly accessible at https://ngdc.cncb.ac.cn/gsa. The whole-genome sequence data reported in this study have been deposited in the Genome Warehouse [49] at the NGDC, BIG, CAS / CNCB [49] (GWH: GWHBFXW01000000–GWHBGBG01000000), and are publicly accessible at https://ngdc.cncb.ac.cn/gwh.

## CRediT author statement

**Cuidan Li:** Validation, Formal analysis, Writing - original draft, Resources, Visualization. **Xiaoyuan Jiang:** Validation, Resources. **Tingting Yang:** Validation, Formal analysis, Resources, Visualization. **Yingjiao Ju:** Validation, Resources. **Zhe Yin:** Resources. **Liya Yue:** Validation, Resources. **Guannan Ma:** Validation, Resources. **Xuebing Wang:** Validation, Resources. **Ying Jing:** Resources. **Xinhua Luo:** Resources. **Shuangshuang Li:** Visualization. **Xue Yang:** Visualization. **Fei Chen:** Conceptualization, Writing - review & editing, Funding acquisition, Project administration, Supervision. **Dongsheng Zhou:** Conceptualization, Writing - review & editing, Project administration, Supervision. All authors have read and approved the final manuscript.

## Competing interests

The authors declare no competing interests.
