# Supplementary material for: Genomic Epidemiology of Carbapenemase-producing Klebsiella pneumoniae in China
Source: Genomics Proteomics Bioinformatics. 2022 Mar 18;20(6):1154–67. doi: 10.1016/j.gpb.2022.02.005 (PMC10225488; doi:10.1016/j.gpb.2022.02.005)
Supplement: Supplementary data 3 [file mmc3.docx]

**Table S3 Carbapenemase genes from our 420 cpKP isolates**

| **Carbapenemase gene** | **Number of isolates (percentage, %)** |
| --- | --- |
| *bla*_KPC_ | 372 (88.57) |
| *bla*_KPC-2_ | 369 (87.86) |
| *bla*_KPC-3_ | 1 (0.24) |
| *bla*_KPC-5_ | 2 (0.48) |
| *bla*_NDM_ | 26 (6.19) |
| *bla*_NDM-1_ | 25 (5.95) |
| *bla*_NDM-5_ | 1 (0.24) |
| *bla*_IMP_ | 19 (4.52) |
| *bla*_IMP-4_ | 13 (3.10) |
| *bla*_IMP-38_ | 6 (1.43) |
| *bla*_KPC_+*bla*_NDM_ | 3 (0.71) |
| *bla*_KPC-2_+*bla*_NDM-1_ | 3 (0.71) |
