# Supplementary material for: Genomic Epidemiology of Carbapenemase-producing Klebsiella pneumoniae in China
Source: Genomics Proteomics Bioinformatics. 2022 Mar 18;20(6):1154–67. doi: 10.1016/j.gpb.2022.02.005 (PMC10225488; doi:10.1016/j.gpb.2022.02.005)
Supplement: Supplementary data 4 [file mmc4.docx]

**Table S4 The distribution of carbapenemase genes from different STs/CGs of 420 cpKP isolates**

| CG | ST | Total  n | *bla*_KPC_–carrying cpKPs  n (%) | *bla*_NDM_–carrying cpKPs  n (%) | *bla*_KPC_- and *bla*_NDM_–carrying cpKPs  n (%) | *bla*_IMP_–carrying cpKPs  n (%) |
| --- | --- | --- | --- | --- | --- | --- |
| CG258 | ST11 | 298 | 295 (99) |  | 1 (0.3) | 2 (0.7) |
|  | ST2667 | 7 | 7 (100) |  |  |  |
|  | ST3348 | 6 | 6 (100) |  |  |  |
|  | ST258 | 2 | 1 (50.0) | 1 (50.0) |  |  |
| CG1 | ST1 | 6 | 2 (33.3) | 4 (66.7) |  |  |
| CG12 | ST12 | 1 | 1 (100) |  |  |  |
| CG13 | ST13 | 1 | 1 (100) |  |  |  |
| CG15 | ST15 | 17 | 16 (94.1) |  |  | 1 (5.9) |
|  | ST2237 | 6 | 6 (100) |  |  |  |
|  | ST3349 | 1 |  | 1 (100) |  |  |
|  | ST14 | 1 |  |  |  | 1 (100) |
| CG17 | ST17 | 3 |  | 1 (33.3) |  | 2 (66.7) |
|  | ST16 | 1 | 1 (100) |  |  |  |
| CG22 | ST1010 | 1 | 1 (100) |  |  |  |
| CG23 | ST23 | 2 | 2 (100) |  |  |  |
|  | ST846 | 1 |  |  |  | 1 (100) |
|  | ST218 | 1 | 1 (100) |  |  |  |
| CG35 | ST449 | 2 |  |  | 2 (100) |  |
| CG37 | ST37 | 4 | 2 (50.0) | 1 (25.0) |  | 1 (25.0) |
|  | ST896 | 1 | 1 (100) |  |  |  |
| CG40 | ST40 | 1 |  | 1 (100) |  |  |
| CG43 | ST43 | 1 |  |  |  | 1 (100) |
| CG45 | ST1493 | 1 |  | 1 (100) |  |  |
|  | ST1106 | 1 | 1 (100) |  |  |  |
| CG65 | ST65 | 2 | 2 (100) |  |  |  |
|  | ST685 | 1 | 1 (100) |  |  |  |
| CG86 | ST86 | 1 | 1 (100) |  |  |  |
| CG147 | ST147 | 7 | 2 (28.6) | 5 (71.4) |  |  |
|  | ST273 | 4 |  | 4 (100) |  |  |
| CG231 | ST231 | 1 | 1 (100) |  |  |  |
| CG292 | ST3345 | 2 |  |  |  | 2 (100) |
| CG307 | ST307 | 7 | 1 (14.3) |  |  | 6 (85.7) |
| CG313 | ST313 | 1 |  | 1 (100) |  |  |
| CG395 | ST395 | 1 | 1 (100) |  |  |  |
| CG515 | ST2176 | 1 |  |  |  | 1 (100) |
| CG661 | ST661 | 1 | 1 (100) |  |  |  |
| CG716 | ST1958 | 5 | 5 (100) |  |  |  |
|  | ST3334 | 1 | 1 (100) |  |  |  |
| CG815 | ST268 | 5 | 5 (100) |  |  |  |
| CG1306 | ST1306 | 1 |  | 1 (100) |  |  |
| CG2390 | ST3350 | 1 |  | 1 (100) |  |  |
| CG2670 | ST2670 | 1 |  | 1 (100) |  |  |
| CG3132 | ST290 | 6 | 5 (83.3) | 1 (16.7) |  |  |
| CG1801 | ST1333 | 1 | 1 (100) |  |  |  |
| NA | ST2480 | 1 |  | 1 (100) |  |  |
| NA | ST3333 | 1 |  | 1 (100) |  |  |
| NA | ST1114 | 1 |  |  |  | 1 (100) |
| NA | ST2153 | 1 | 1 (100) |  |  |  |
