# Supplementary material for: Genomic Epidemiology of Carbapenemase-producing Klebsiella pneumoniae in China
Source: Genomics Proteomics Bioinformatics. 2022 Mar 18;20(6):1154–67. doi: 10.1016/j.gpb.2022.02.005 (PMC10225488; doi:10.1016/j.gpb.2022.02.005)
Supplement: Supplementary data 5 [file mmc5.docx]

**Table S5 Inc groups of the 377 *bla*_KPC_-carrying plasmids**

| Inc group  (*n* = 32) | Number of plasmids  (*n* = 377) | Percentage (%) |
| --- | --- | --- |
| IncFII_pHN7A8_ | 28 | 7.43 |
| IncFII_pHN7A8_:IncR | 163 | 43.24 |
| IncFII_pHN7A8_:Inc_pA1763-KPC_ | 59 | 15.65 |
| IncFII_pHN7A8_:IncN1 | 1 | 0.27 |
| IncFII_pHN7A8_:IncN1:IncR | 2 | 0.53 |
| IncFII_pHN7A8_:Inc_pA1763-KPC_:IncN1 | 2 | 0.53 |
| IncFII_pHN7A8_:IncFIB_pFB2.3_ | 1 | 0.27 |
| IncFII_pKPHS2_:Inc_pA1763-KPC_ | 36 | 9.55 |
| IncFII_pKPHS2_:IncR | 5 | 1.33 |
| IncFII_pKPHS2_:IncFIB_pSC138_ | 1 | 0.27 |
| IncFII_pKPHS2_:Inc_pLT968725_ | 1 | 0.27 |
| IncFII_pKPHS2_:Inc_pA1763-KPC_:IncR | 2 | 0.53 |
| IncFII_p0716-KPC_:Inc_pA1763-KPC_ | 30 | 7.96 |
| IncFII_p0716-KPC_:IncR | 1 | 0.27 |
| IncFII_p0716-KPC_:IncFIB_pSC138_ | 1 | 0.27 |
| IncFII_pCP020359_:Inc_pA1763-KPC_ | 7 | 1.86 |
| IncFII_R100_ | 4 | 1.06 |
| IncFII_R100_:IncFIB_plasmid F_ | 1 | 0.27 |
| IncFII_R100_:IncFIA_pBK30661_ | 1 | 0.27 |
| IncFII_pBK30683_ | 1 | 0.27 |
| IncX6 | 10 | 2.65 |
| IncR | 3 | 0.80 |
| IncR:IncFIB_plasmid F_ | 1 | 0.27 |
| IncR:Inc_pA1763-KPC_:IncN1 | 2 | 0.53 |
| IncP-6 | 3 | 0.80 |
| IncC | 2 | 0.53 |
| IncC:IncR | 1 | 0.27 |
| IncN1 | 1 | 0.27 |
| IncFIA_pBK30661_ | 2 | 0.53 |
| Inc_pA1763-KPC_ | 2 | 0.53 |
| Inc_pHS062105-3_ | 2 | 0.53 |
| IncFIB_pSC138_ | 1 | 0.27 |

*Note*: These 377 plasmids came from a total of 375 cpKP isolates. There were two different *bla*_KPC_-carrying plasmids in each of the two following isolates: an IncFII_pHN7A8_:IncR plasmid plus an IncX6 one in the G030 isolate, and an IncFII_R100_:IncFIA_pBK30661_ plasmid plus an IncR:IncFIB_plasmid F_ one in the G300 isolate.
