# Supplementary material for: Genomic Epidemiology of Carbapenemase-producing Klebsiella pneumoniae in China
Source: Genomics Proteomics Bioinformatics. 2022 Mar 18;20(6):1154–67. doi: 10.1016/j.gpb.2022.02.005 (PMC10225488; doi:10.1016/j.gpb.2022.02.005)
Supplement: Supplementary data 6 [file mmc6.docx]

**Table S6 The Inc groups of *bla*_KPC_-carrying plasmids of the 38 complete ST11 KP genomes downloaded from NCBI**

| **Inc group** | **Number** | **Percentage (%)** |
| --- | --- | --- |
| IncFII_pHN7A8_ | 3 | 7.5 |
| IncFII_pHN7A8_:IncN1 | 1 | 2.5 |
| IncFII_pHN7A8_:IncR | 27 | 67.5 |
| IncFII_pHN7A8_:IncR:IncN1 | 1 | 2.5 |
| IncFII_pHN7A8_:Inc_pA1763-KPC_ | 1 | 2.5 |
| IncFII_pHN7A8_:IncΔR | 1 | 2.5 |
| IncFII_K_:IncR | 2 | 5 |
| IncFII_K_:IncR:Inc_pA1763-KPC_ | 1 | 2.5 |
| IncR | 3 | 7.5 |
