# Supplementary material for: Genomic Epidemiology of Carbapenemase-producing Klebsiella pneumoniae in China
Source: Genomics Proteomics Bioinformatics. 2022 Mar 18;20(6):1154–67. doi: 10.1016/j.gpb.2022.02.005 (PMC10225488; doi:10.1016/j.gpb.2022.02.005)
Supplement: Supplementary data 7 [file mmc7.docx]

**Table S7 Percentage of cpKP isolates resistant to the 21 antibiotics in China**

| **Class** | **Antibiotics** | **Percentage (%)** | **PMID** |
| --- | --- | --- | --- |
| Penicillins | Ampicillin | 99.85 | 30426083, 29855274, 28560183, 29997928, 29558475, 31682180, 28659886, 28594635, 28705769, 31734749 |
|  | Ampicillin/sulbactam | 95.61 | 28693691, 28560183, 29873627, 29558475, 31682180, 28659886, 30603949, 30783127 |
|  | Piperacillin | 99.08 | 28693691, 28659886 |
|  | Piperacillin/tazobactam | 92.57 | 32042732, 30588043, 30481921, 30426083, 28560183, 31050634, 29997928, 29873627, 29558475, 31682180, 31334685, 28594635, 30603949, 30783127, 30423053, 31433255, 31533609, 31734749 |
| Cephalosporins, first generation | Cefazolin | 99.14 | 32042732, 30481921, 30426083, 28560183, 31050634, 29997928, 29873627, 29558475, 31682180, 28659886, 28594635, 30783127, 31734749 |
| Cephalosporins, second generation | Cefuroxime | 98.24 | 30498365, 29997928, 28659886 |
|  | Cefotetan | 93.74 | 30481921, 28560183, 29997928, 29558475, 31682180, 30783127 |
| Cephalosporins, third generation | Ceftazidime | 96.30 | 28693691, 30588043, 30481921, 30498365, 29855274, 28560183, 29997928, 28643488, 29558475, 31682180, 28659886, 28594635, 30603949, 30783127, 28705769, 30423053, 31533609, 28388651 |
|  | Ceftriaxone | 94.56 | 32042732, 30481921, 28560183, 31050634, 29997928, 28643488, 29873627, 29558475, 31682180, 31334685, 30603949, 30783127, 31533609, 31734749 |
| Cephalosporins, fourth generation | Cefepime | 89.19 | 32042732, 28693691, 30588043, 30481921, 30426083, 29855274, 28560183, 31050634, 29997928, 28643488, 29873627, 31682180, 28659886, 31334685, 28594635, 30603949, 30783127, 28705769, 28450933, 31533609, 28388651, 31734749 |
| Monobactams | Aztreonam | 91.91 | 28693691, 30588043, 30481921, 30498365, 28560183, 31050634, 29558475, 31682180, 28659886, 31334685, 28594635, 30603949, 30783127, 28705769, 30423053, 31433255, 31533609, 28388651, 31734749 |
| Carbapenems | Impenem | 91.42 | 32042732, 28693691, 30588043, 30481921, 30498365, 30426083, 29855274, 28560183, 31050634, 29997928, 29873627, 29558475, 31682180, 28659886, 31334685, 28594635, 30603949, 28705769, 30423053, 28450933, 31433255, 31533609, 28388651 |
|  | Meropenem | 86.51 | 32042732, 28693691, 30588043, 30481921, 30498365, 30426083, 29855274, 31735038, 29997928, 31682180, 28659886, 31365977, 28594635, 30603949, 31921701, 28705769, 30423053, 28450933, 31433255, 28388651 |
| Aminoglycosides | Amikacin | 49.45 | 32042732, 28693691, 30588043, 30498365, 30426083, 29855274, 30529506, 28560183, 31735038, 31050634, 29997928, 29873627, 29558475, 31682180, 28659886, 31334685, 28594635, 30603949, 31921701, 30783127, 28705769, 30423053, 28450933, 31433255, 31533609, 28388651 |
|  | Gentamicin | 64.72 | 32042732, 28693691, 30481921, 30498365, 29855274, 30529506, 28560183, 31050634, 29997928, 28643488, 29873627, 29558475, 31334685, 28594635, 30603949, 30783127, 28705769, 30423053, 28450933, 31433255, 31533609, 28388651 |
|  | Tobramycin | 71.54 | 28693691, 30529506, 28560183, 31050634, 28643488, 29558475, 30783127, 31433255 |
| Fluoroquinolones | Ciprofloxacin | 78.25 | 32042732, 30588043, 30481921, 30498365, 30426083, 29855274, 30529506, 28560183, 31050634, 29997928, 28643488, 29558475, 31682180, 28659886, 31334685, 28594635, 30603949, 30783127, 28705769, 30423053, 31433255, 31533609, 28388651 |
|  | Levofloxacin | 68.17 | 32042732, 30481921, 30498365, 30529506, 28560183, 31735038, 31050634, 29997928, 28643488, 29873627, 29558475, 31682180, 31334685, 28594635, 30603949, 30783127, 31433255, 31533609, 28388651 |
| Furanes | Macrodantin | 95.70 | 31050634 |
| Sulfanilamides | Sulfamethoxazole/trimethoprim | 70.22 | 30481921, 30498365, 30426083, 29855274, 30529506, 28560183, 31050634, 29873627, 29558475, 28659886, 28594635, 30783127, 28705769, 30423053, 31433255, 31533609, 28388651, 31734749 |

*Note*: Data are derived from all the 31 literatures published since 2017.
