# Supplementary material for: Genomic Epidemiology of Carbapenemase-producing Klebsiella pneumoniae in China
Source: Genomics Proteomics Bioinformatics. 2022 Mar 18;20(6):1154–67. doi: 10.1016/j.gpb.2022.02.005 (PMC10225488; doi:10.1016/j.gpb.2022.02.005)
Supplement: Supplementary data 9 [file mmc9.docx]

**Table S9 PCR primers used in the competition experiment**

| **cpKP isolate** | **ST/CG** | ***bla*_KPC_-carrying plasmid** | **Target gene** | **Primer sequence** |
| --- | --- | --- | --- | --- |
| G134 | ST11/CG258 | IncFII_pHN7A8_ | G134_05212 | F: 5'-ACCGAAACATTCTCCGCACT-3' |
|  |  |  |  | R: 5'-CCTGCGGAAACAACCTGGTA-3' |
| G285 | ST11/CG258 | IncFII_pHN7A8_:IncR | G285_01367 | F: 5'-CGCTCTGAGAACGTCGTCAT-3' |
|  |  |  |  | R: 5'-ACCTGGAAATGCGGGTCTTT-3' |
| G318 | ST11/CG258 | IncFII_pHN7A8_:Inc_pA1763-KPC_ | G318_02254 | F: 5'-CTCATCCATCGCACTACCCG-3' |
|  |  |  |  | R: 5'-AGGGTAGGTGAAAAGCTCGC-3' |
| G165 | ST11/CG258 | IncFII_p0716-KPC_:Inc_pA1763-KPC_ | G165_02217 | F: 5'-TTACAAGGGCCGCTGACATT-3' |
|  |  |  |  | R: 5'-CGGGTAGTGCGATGGATGAG-3' |
| G344 (control) | Non-CG258 | IncFII_pKPHS2_:Inc_pA1763-KPC_ | G344_00764 | F: 5'-TTGCCTTTCAGATCGCGACT-3' |
|  |  |  |  | R: 5'-GTCTCAGGGCCATCAGTAGC-3' |

*Note*: Each isolate contained a single plasmid.
